# Intra- and Interspecies Genomic Transfer of the *Enterococcus faecalis* Pathogenicity Island

**DOI:** 10.1371/journal.pone.0016720

**Published:** 2011-04-29

**Authors:** Jenny A. Laverde Gomez, Antoni P. A. Hendrickx, Rob J. Willems, Janetta Top, Irina Sava, Johannes Huebner, Wolfgang Witte, Guido Werner

**Affiliations:** 1 Wernigerode Branch, Robert Koch Institute, Wernigerode, Germany; 2 Department of Medical Microbiology, University Medical Center Utrecht, Utrecht, The Netherlands; 3 Division of Infectious Diseases, Department of Medicine, Freiburg University Hospital, Freiburg, Germany; Charité-University Medicine Berlin, Germany

## Abstract

Enterococci are the third leading cause of hospital associated infections and have gained increased importance due to their fast adaptation to the clinical environment by acquisition of antibiotic resistance and pathogenicity traits. *Enterococcus faecalis* harbours a pathogenicity island (PAI) of 153 kb containing several virulence factors including the enterococcal surface protein (*esp*). Until now only internal fragments of the PAI or larger chromosomal regions containing it have been transfered. Here we demonstrate precise excision, circularization and horizontal transfer of the entire PAI element from the chromosome of *E. faecalis* strain UW3114. This PAI (ca. 200 kb) contained some deletions and insertions as compared to the PAI of the reference strain MMH594, transferred precisely and integrated site-specifically into the chromosome of *E. faecalis* (intergenic region) and *Enterococcus faecium* (tRNA*lys*). The internal PAI structure was maintained after transfer. We assessed phenotypic changes accompanying acquisition of the PAI and expression of some of its determinants. The *esp* gene is expressed on the surface of donor and both transconjugants. Biofilm formation and cytolytic activity were enhanced in *E. faecalis* transconjugants after acquisition of the PAI. No differences in pathogenicity of *E. faecalis* were detected using a mouse bacteraemia and a mouse peritonitis models (tail vein and intraperitoneal injection). A 66 kb conjugative pheromone-responsive plasmid encoding *erm*(B) (pLG2) that was transferred in parallel with the PAI was sequenced. pLG2 is a pheromone responsive plasmid that probably promotes the PAI horizontal transfer, encodes antibiotic resistance features and contains complete replication and conjugation modules of enterococcal origin in a mosaic-like composition. The *E. faecalis* PAI can undergo precise intra- and interspecies transfer probably with the help of conjugative elements like conjugative resistance plasmids, supporting the role of horizontal gene transfer and antibiotic selective pressure in the successful establishment of certain enterococci as nosocomial pathogens.

## Introduction

Enterococci are increasingly important nosocomial pathogens, known for being highly recombinant and for possessing several antibiotic resistance traits. These characteristics provide them with the capacity to adapt to clinical environments and modify their pathogenic properties thereby representing the possibility of acquisition and transfer of these genes from and to other pathogens. The *Enterococcus faecalis* Pathogenicity island (PAI) (153 kb) was first described by Shankar *et al.*. It encodes several pathogenicity factors, among them the enterococcal surface protein (*esp*) conferring increased biofilm and colonization, a cytolysin with haemolytic, cytolytic and antibacterial activity, the aggregation substance, a bile acid hydrolase, surface proteins and general stress proteins [1]. The *E. faecalis* PAI is widely distributed among isolates of different origins, clonal types and complexes, and it probably evolved by modular gain and loss of internal gene clusters [2]. Contrasting the situation in Gram-negatives, where PAI structures are not modular and quite conserved, the *E. faecalis* PAI shows a highly variable gene content in isolates of different origins and regions [3], [4], [2], [5]. The mechanisms underlying the mobilization and transfer of PAIs are still not well understood, neither is the evolution of PAIs within a certain bacterial species. The *E. faecalis* PAI in MMH594 has a higher G + C content compared to the chromosome, contains phage-related integrase and excisionase genes, and 10 bp Direct Repeats (DR) at the flanking ends [1] that resemble the *attL* and *attR* of integrated DNA. These characteristics suggest that the PAI can be mobilized as an ICE [6], [7]. For ICEs mobilization, the excisionase- mediated homologous recombination between *attL* and *attR* produces excision and formation of circular intermediates (carrying *attB*), while a recombinase (integrase) catalyzes integration by recombination between *attB* and a chromosomal target sequence *attP* generating the junction sequences *attL* and *attR* [8].

The spontaneous excision from the chromosome and formation of circular intermediates are supposed to be prerequisites for the horizontal transfer of PAIs and have been observed in the PAIs of *Vibrio* [9], [10], *Salmonella* [11], *Yersinia pseudotuberculosis* [12], *Shigella flexneri* and *Escherichia coli* [13], [14].

Horizontal transfer of PAIs into recipient strains facilitated by phages has been described in VPI-1 [15] and SaPI-1 [16]. Similarly, plasmids harbouring regions of three SaPIs, of the *E. coli* O26 locus of enterocyte effacement (LEE O26) and of *she*PAI demonstrated PAI integration into the chromosome of recipient strains [17], [18], [12], while SGI1 and the *E. coli* HPI have been shown to transfer with the help of plasmids [11], [19]. So far, only the *Y. tuberculosis* HPI has been observed to transfer spontaneously in a liquid medium without the help of a phages or plasmids [20]. Previous attempts to transfer precisely the *E. faecalis* PAI have failed; only parts of the PAI or larger chromosomal elements containing the PAI have been observed to transfer [21], [6], [7]. Here we confirm for the first time precise excision, circularization and horizontal transfer of the entire *E. faecalis* PAI and integration into the *E. faecium* and *E. faecalis* chromosomes in parallel with the conjugative transfer of the *erm*(B) -encoding pheromone-plasmid pLG2. The acquisition of the PAI modulated certain pathogenic properties such as *esp* expression, cytolytic activity and biofilm formation. These findings support the role of horizontal gene transfer in the evolution of enterococci and transformation from commensal to pathogenic variants under antibiotic selective pressure.

## Results

### Transfer of the *E. faecalis* PAI

The *esp* gene was transferred from *E. faecalis* donor strain UW3114 into *E. faecalis* and *E. faecium* recipients by filter mating conjugation along with the transfer of plasmid- encoded erythromycin resistance.

**Figure 1:**
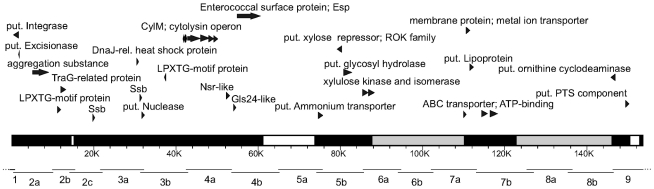
The *E. faecalis* PAI of strain UW3114 that was horizontally transfered. The structure was investigated based on the *E. faecalis* PAI of strain MMH594 (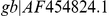), by long template PCR, regular PCR, sequencing and Southern hybridizations. The long template PCR- regions are indicated by horizontal lines (1 to 9) (Table S1). Regions present are shown in black and sum up to *ca.* 90.6 kb. White regions are absent. Grey regions are absent and hold additional insertions. Only representative ORFs from the regions present are shown.

PCR analysis of overlapping regions covering the entire PAI (153.57 kb as in reference strain MMH594) demonstrated that the transferred PAI of donor strain UW3114 was different to the PAI in the reference strain MMH594. However, no difference was observed between the donor and the transconjugants, indicating that the PAI did not undergo any changes during horizontal transfer (Figure 1, Table 1). Approximately 90.6 kb of the reference PAI -MMH594 were present in the PAI of UW3114 including the aggregation substance gene, the complete cytolysin operon and the entire *esp* gene. Long PCR bridging the gaps of the putative absent regions confirmed deletions located in regions: 2b-2c, 4b-5a and 9 (Figure 1, Table S1). No regular or long PCR amplification was possible that closed the gaps in regions 6a-6b and 7b-8b, suggesting that elements too large to be amplified were present (Figure 1). PCR at the flanking ends of the PAI confirmed exact presence of the PAI ends integrated into the chromosome of *E. faecalis* and *E. faecium* (see below).

**Table 1:** Results of Long template PCRs and regular PCRs screening for presence of the entire *E. faecalis* PAI.

Long PCR-region	MMH594	UW3114	64/3x UW3114 T-10	OG1RFx UW3114 T-12	Regular PCR - ORF	MMH594	UW3114	64/3x UW3114 T-10	OG1RFx UW3114 T-12
1	+	+	-	+	EF0001	+	+	+	+
2a	+	+	+	+	EF0005	+	+	+	+
2b 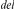	+	-	-	-	EF0009	+	+	+	+
					EF0011	+	+	+	+
					EF0012	+	-	-	-
2c 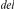	+	+ *	+ *	+ *	EF0013	+	+	+	+
					EF0014	+	+	+	+
					EF0015	+	+	+	+
					EF0016	+	+	+	+
					EF0017	+	+	+	+
					EF0019	+	+	+	+
					EF0020	+	+	+	+
					EF0021	+	+	+	+
3a	+	+	+	+					
									
3b	+	+	+	+					
									
4a	+	+	+	+	EF0046	+	+	+	+
4b 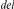	+	-	+ *	+ *	EF0055	+	+	+	+
					EF0056	+	+	+	+
					EF0057	+	-	-	-
					EF0058	+	-	-	-
					EF0059	+	-	-	-
					EF0060	+	-	-	-
5a 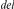	+	-	-	-	EF0061	+	-	-	-
					EF0062	+	-	-	-
					EF0065	+	-	-	-
					EF0066	+	-	-	-
					EF0067	+	-	-	-
					EF0068	+	-	-	-
					EF0069	+	-	-	-
					EF0070	+	-	-	-
					EF0072	+	+	+	+
					EF0073	+	+	+	+
5b	+	+	+	+	EF0074	+	+	+	+
					EF0076	+	+	+	+
					EF0077	+	+	+	+
					EF0078	+	+	+	+
					EF0079	+	+	+	+
					EF0080	+	+	+	+
					EF0081	+	+	+	+
					EF0082	+	+	+	+
6a 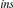	+	-	-	-	EF0083	+	+	+	+
					EF0084	+	-	-	-
6b 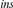	+	-	-	-	EF0087	+	-	-	-
					EF0089	+	-	-	-
					EF0091	+	-	-	-
7a 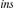	+	-	-	-	EF0092	+	-		-
					EF0093	+	+	+	+
					EF0094	+	+	+	+
					EF0095	+	+	+	+
7b	+	+ *	+ *	+ *	EF0101	+	+	+	+
					EF0102	+	+	+	+
					EF0108	+	+	+	+
8a 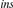	+	-	-	-	EF0109	+	-	-	-
					EF0111	+	-	-	-
					EF0115	+	-	-	-
8b 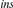	+	-	-	-	EF0117	+	-	-	-
					EF0119	+	-	-	-
					EF0122	+	-	-	-
					EF0123	+	-	-	-
9 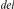	+	+*	-	+*	EF0124	+	+	+	+
					EF0125	+	+	+	+
					EF0126	+	+	+	+
					EF0126	+	+	+	+
					EF0128	+	+	+	+
					EF0128-129	+	-	-	-
					EF0129	+	-	-	-
					TSP2 3′-9PAIR	+	+	-	+

Presence of the entire PAI was investigated based on reference strain MMH594 [1]) in the donor and transconjugant strains PAI overlapping regions were arbitrarly chosen to allow long-PCR amplification as shown in Table S1 and Figure 1. Recipient strains were also tested and were negative for all PCRs.

* A product of unexpected size was amplified. 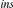 an insertion within this region(s) is suspected. 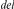 a deletion within this region(s) was confirmed.

**Figure 2:**
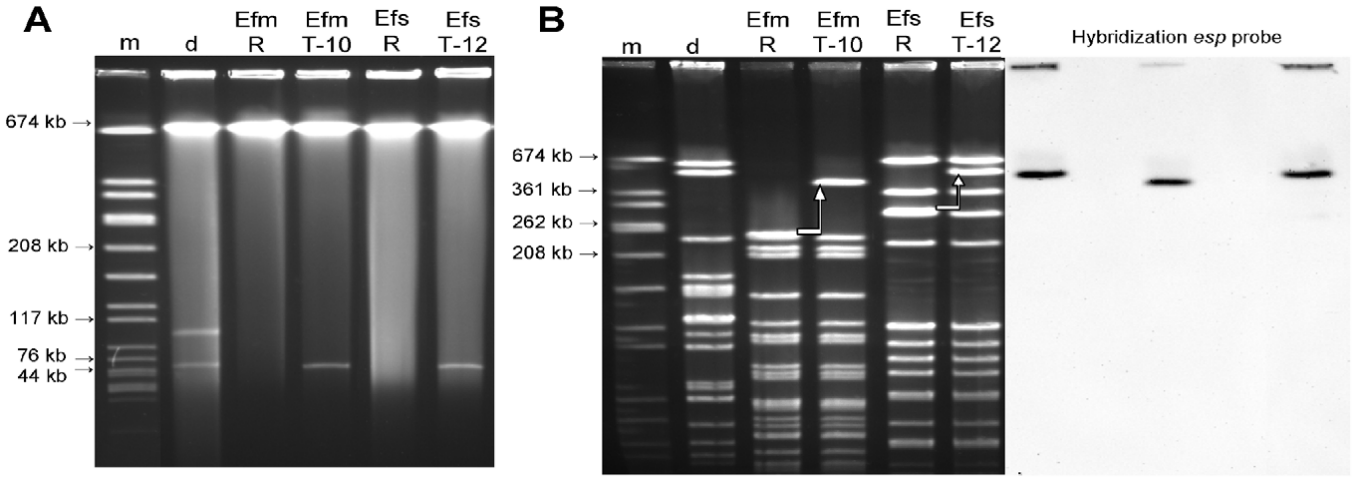
A. S1-nuclease analysis showing transfer of a *ca.* 66 kb conjugative plasmid into both transconjugants. B. *Sma*I restriction analysis and corresponding Southern hybridization using an *esp* probe. None of the plasmid bands hybridized to an *esp* probe (not shown). M, Marker *S. aureus* 8325 *Sma*I -digested; D, Donor UW3114; Efm-R, *E. faecium* recipient 64/3; Efm T-10, *E. faecium* transconjugant 64/3xUW3114 T-10; Efs-R, *E. faecalis* recipient OG1RF; Efs T-12, *E. faecalis* transconjugant OG1RFxUW3114 T-12. White arrows indicate the fragment size shift due to PAI acquisition in *E. faecalis* (*ca.* 206 kb larger) and *E. faecium* (*ca.* 193 kb larger).

The size of the transferred PAI element and its chromosomal integration were determined by Pulse field gel electrophoresis (PFGE) and Southern hybridization. *Sma*I - PFGE analysis revealed that a single restriction fragment in each transconjugant strain was enlarged and hybridised to the *esp* probe (Figure 2). The fragment size shift appeared to be *ca.* 200 kb (calculated sizes were 206 kb in *E. faecalis* and 193 kb in *E. faecium*) and corresponded to the total size of the PAI transferred element. Given that only 90.6 kb of the reference PAI were confirmed to be present and that additional DNA seemed to be integrated within certain regions (see above), it is likely that additional *ca.* 119.4 kb DNA were inserted within the PAI (Figure 1). S1-nuclease analysis demonstrated that the *E. faecalis* and *E. faecium* transconjugants acquired a ca. 66 kb plasmid (pLG2) (Figure 2), which did not hybridize to the *esp* probe, but to the selection marker *erm*(B). I-*Ceu*I macrorestriction analysis confirmed localization of *esp* on a chromosomal band in both transconjugant strains (data not presented).

### Integration site

**Figure 3:**
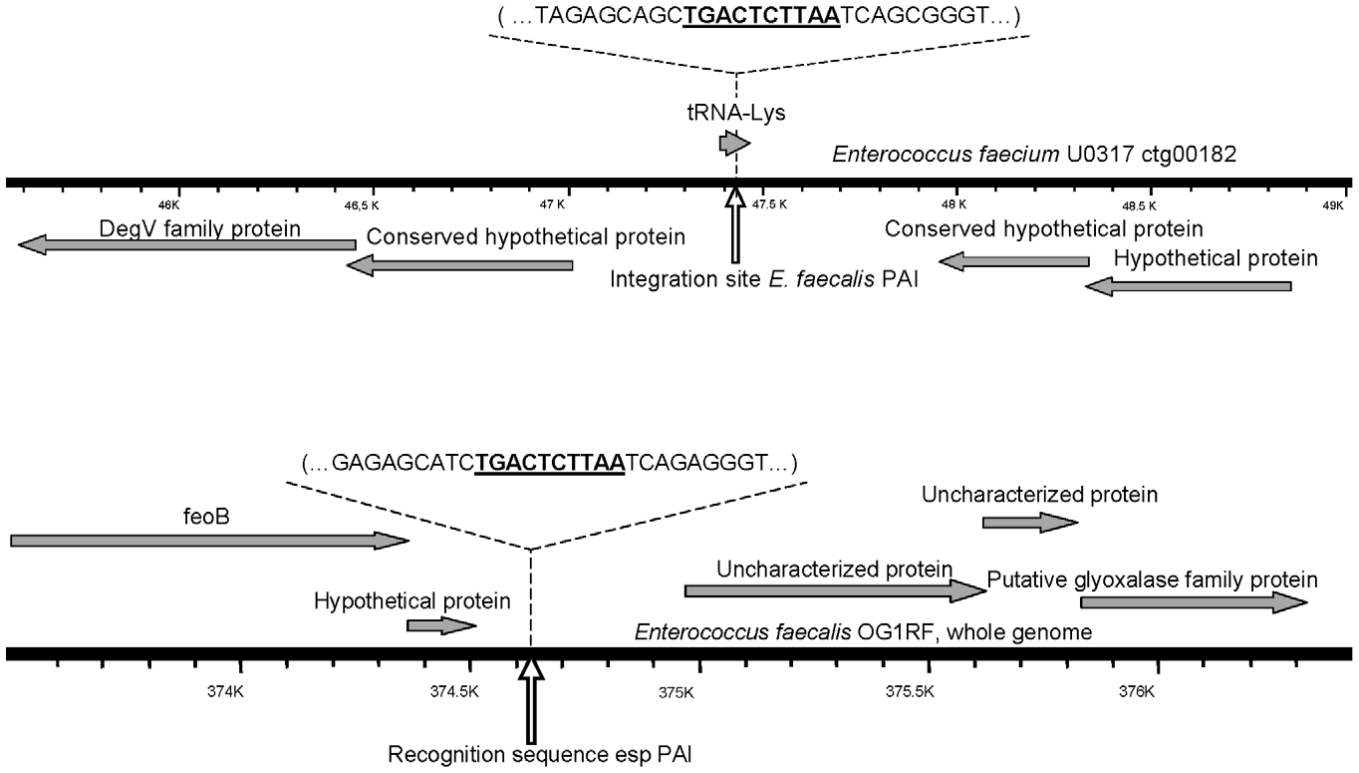
Chromosomal region where the *E. faecalis* PAI integrated in *E. faecium* as in strain U0317 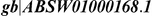 (top) and *E. faecalis* OG1RF 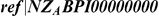 (bottom). The vertical white arrow points at the integration site of the *E. faecalis* PAI. The detailed nucleotide sequence shows the 10 bp region of recognition and integration of the PAI as underlined bold text.

**Figure 4:**
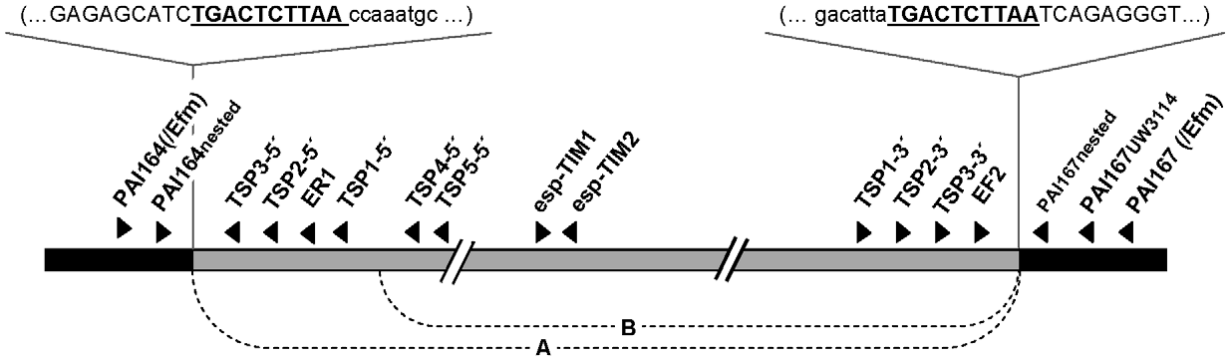
Representation of the *E. faecalis* PAI showing the primers used in this study for *esp* detection and investigation of PAI excision, circularization and integration (not to scale). Arrowheads represent the primers: white arrowheads are primers used to investigate excision from the chromosome, and grey arrowheads are primers used to detect circular intermediates. Solid black region represents chromosomal DNA, grey region represents the PAI. The junction- nucleotide sequences after PAI integration in the chromosome of *E. faecalis* are shown in detail; the DR appear in underlined bold text. Dashed lines indicate joining of PAI regions during formation of circular intermediates; A and B and precise excision indicate the different events of excision and circularization of the PAI that were confirmed in this study.

The integration site of the *E. faecalis* PAI in the chromosome of *E. faecalis* strain OG1RF (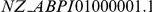) is at coordinates 374617∶374626 (Figure 3). This integration site was found by comparing the OG1RF genome to the chromosomal region spanning the *E. faecalis* PAI in the genome of *E. faecalis* strain V583 (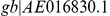) (PAI coordinates, 445126∶583433). PCR amplification of the integration site of the PAI prior to its insertion confirmed that it is intact in the recipient strain OG1RF and occupied by the PAI in the transconjugant OG1RFxUW3114 T-12. The region where the PAI integrated into the *E. faecium* transconjugant 64/3xUW3114 T-10 genome was identified to be a tRNA*lys* gene, which is located in the *E. faecium* U0317 contig00182 (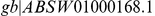) at coordinates 47388∶47460 (Figure 3). PCR amplification confirmed that the identified integration site tRNA*lys* was intact in the recipient strain 64/3 and occupied by the *E. faecalis* PAI in the transconjugant 64/3xUW3114 T-1 (Figure 4).

BLAST comparisons of the *E. faecalis* and the *E. faecium* regions where the *E. faecalis* PAI integrated revealed that they share 85% similarity at the nucleotide level along a 94 and 93 bp region respectively. A 10 bp (AATTCTCAGT) sequence (resembling *attB*) was found to be present at the PAI integration site of the *E. faecium* and *E. faecalis* recipient strains. PCR and sequencing of the flanking ends of the PAI and chromosomal regions spanning it demonstrated the exact integration of the PAI and confirmed the presence of the two 10 bp DR (resembling *attl* and *attR*).

The region where the *E. faecium* putative *esp* PAI is located in *E. faecium* (*E. faecium* U0317 Contig 00248 (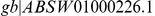) [22]) was amplified by PCR and confirmed to be free in the recipient (64/3) and transconjugant (64/3xUW3114 T-10) strains, indicating that neither the *E. faecium* nor the *E. faecalis* PAI are integrated in this chromosomal region in the *E. faecium* donor and transconjugant strains.

### Excision and circularization

Presence of a circular intermediate formed from the precisely excised PAI element could be detected by nested PCR in the donor strain UW3114. Sequencing of these amplicons confirmed that the PAI can precisely excise at its flanking ends and that these ends join together, resulting in circularization of the PAI element (See Fig. 4). The precisely excised circular intermediate carried the specific 10 bp sequence (AATTCTCAGT) resembling *attP*. Accordingly, excision of the PAI from the chromosome could be confirmed by amplification of the free chromosomal region that contained the PAI in the *E. faecalis* donor strain UW3114. Excision from the chromosome could also be detected only after two rounds of nested PCR. In addition to precise excision and circularization of the PAI (only detected in donor UW3114), imprecise PAI excision and circularization was also observed. PCR and sequencing demonstrated that different fragments of the PAI can remain in the chromosome after excision, or chromosomal fragments can be excised together with the PAI. The following two scenarios of imprecise excision were the most frequently detected: (a) the fragment 1∶223 bp of PAI remains in the chromosome after PAI excision and (b) the fragment 1∶1704 bp of PAI remains in the chromosome after PAI excision (Figure 4).

Case (a) was detected in donor strain UW3114, while case (b) was seen in reference strain MMH594, donor strain UW3114 and transconjugants 64/3xUW3114 T-10 and OG1RFxUW3114 T-12. These events of imprecise excision could be triggered by the similarity between internal regions of the PAI at this positions and the chromosomal region flanking downstream the PAI.

### Phenotypic changes after PAI acquisition

The acquisition of the PAI changed some phenotypic properties of the *E. faecium* and *E. faecalis* strains (Table 2). Presence of Esp protein on the cell surface, biofilm formation, cytolytic activity and growth *in vitro* were assessed. Pathogenicity and in vivo survival of the *E. faecalis* strains was compared using mouse bacteraemia and peritonitis models.

**Table 2:** Strains used in this study. *Efm*, *E. faecium*; *Efs E. faecalis*.

Strain	Description	Resistance	PAI present	plasmid pLG2 present
Reference:				
MMH594	*Efs* bears the originally described PAI	GEN/ERY	Yes	-
Donor:				
UW3114	*Efs*	ERY	Yes	Yes
Recipients:				
OG1RF	*Efs*	RAM/FUS	No	No
64/3	*Efm*	RAM/FUS	No	No
Transconjugants:				
OG1RF T-12	*Efs* derived from OG1RF	RAM/FUS/ERY	Yes	Yes
OG1RF T-1*	*Efs* derived from OG1RF	RAM/FUS/ERY	No	Yes
64/3 T-10	*Efm* derived from 64/3	RAM/FUS/ERY	Yes	Yes
64/3 T-1*	*Efm* derived from 64/3	RAM/FUS/ERY	No	Yes

Only antibiotic resistances relevant for filter mating experiments are presented. GEN, gentamicin-high level (>2048 mg/L), RAM, rifampicin (>256 mg/L); FUS, fusidic acid (>16 mg/L); ERY, erythromycin (>8 mg/L).

T1* correspond to the transconjugants that acquired the *erm*(B) -encoding plasmid pLG2 and lack *E. faecalis* PAI.

#### Cell surface *esp* expression

**Figure 5:**
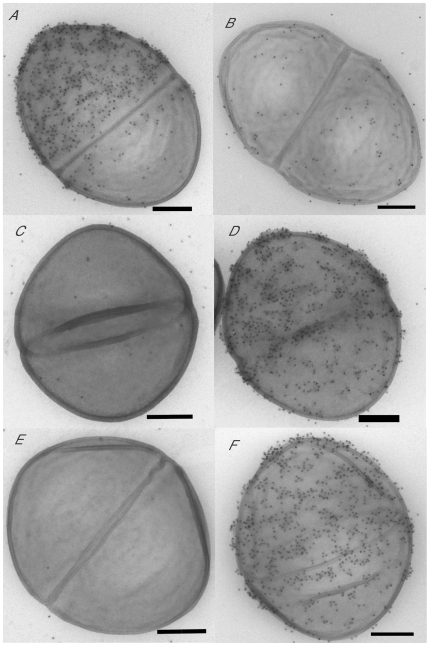
Esp expression shown by transmission electron microscopy of cells negatively stained and labelled with inmunogold (15 nm) using anti-Esp antiserum. Strains UW3114 (donor) and MMH594 (reference) are shown for comparison. 64/3 and OG1RF are the recipient strains. Strains 64/3xUW3114 T-10 and OG1RFxUW3114 T-12 are the transconjugants that acquired the plasmid pLG2 and the *E. faecalis* PAI from strain UW3114 (See also Table 2). Bar lenght = 200 nm.

**Figure 6:**
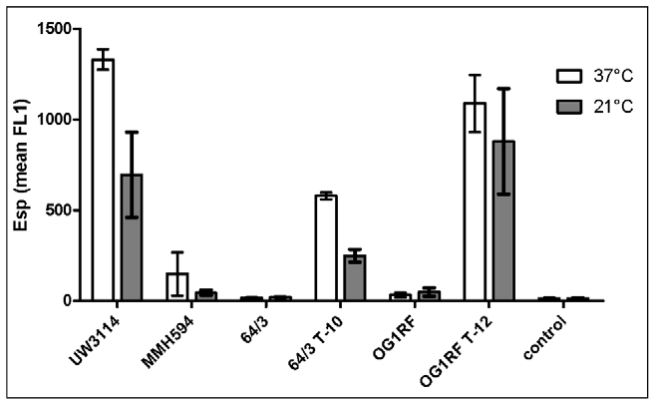
Differential expression of cell wall-associated Esp at 37°C and 21°C measured by flow cytometry (FACS) of anti-Esp labelled cells. Strains UW3114 (donor) and MMH594 (reference) are shown for comparison. 64/3 and OG1RF are the recipient strains. Strains 64/3 T-10 (64/3xUW3114 T-10) and OG1RF T-12 (OG1RFxUW3114 T-12) are the transconjugants that acquired the *E. faecalis* PAI from strain UW3114 (See also Table 2). Notice the enhancement of *esp* expression in the transconjugant strains compared to the corresponding recipient (p<0.05), and decreased *esp* expression at 21°C(p = 0.0076). Control is a conjugate control. The average of the mean values from two different experiments are presented, error bars denote standard deviation.

cTransmission immunoelectron microscopy displayed the Esp protein associated with the cell wall of the donor and transconjugant strains but not on the surfaces of the recipients (Figure 5). FACS analysis revealed no Esp on the cell surface of the recipient strains. Enhanced expression in the *E. faecalis* transconjugant strain OG1RFxUW3114 T-12 (p<0.05) and in the *E. faecium* transconjugant 64/3xUW3114 T-10 (p<0.05), compared to their corresponding recipient strains, was confirmed using one way ANOVA and Tukey's post-test. The *E. faecium* transconjugant 64/3xUW3114 T-10 showed a slightly lower *esp* expression than the *E. faecalis* strain (p>0.05) (Figure 6). All strains had a lower *esp* expression at 21°C in comparison to that at 37°C (p = 0.0076), as confirmed using a two way ANOVA comparisson (Figure 6).

#### Biofilm formation

**Figure 7:**
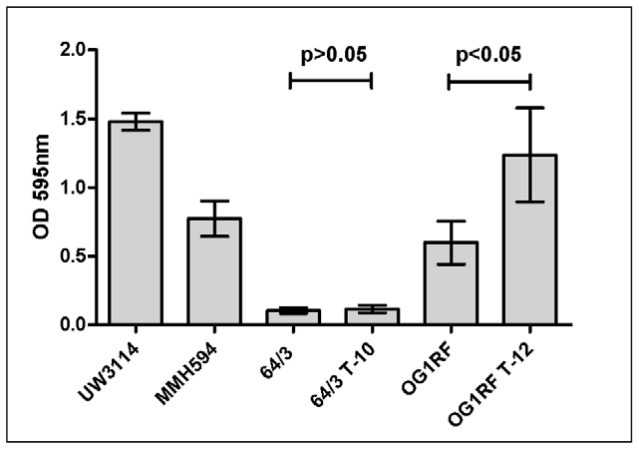
Comparison of *in vitro* biofilm formation. Strains UW3114 (donor) and MMH594 (reference) are shown for comparison. 64/3 and OG1RF are the recipient strains. Strains 64/3 T-10 (64/3xUW3114 T-10) and OG1RF T-12 (OG1RFxUW3114 T-12) are the transconjugants that acquired the plasmid pLG2 and *E. faecalis* PAI from strain UW3114 (See also Table 2). Notice the significant increase in Biofilm formation of PAI positive *E. faecalis* transconjugant. The mean values of three different experiments are shown. Error bars denote standard deviation. Experiments were performed four times, each time in triplicate.

The *E. faecalis* transconjugant strain OG1RFxUW3114 T-12 showed a significant 2-fold increase in biofilm forming capacity compared to its homologous strain, the recipient OG1RF (p<0.05). In the *E. faecium* strains both the recipient and the transconjugants showed a similar level of low biofilm formation (p>0.05) (Figure 7). Statistical significance was evaluated using One way ANOVA with Tukey's post test.

#### Cytolysin/haemolysin

Both the reference (MMH594) and the donor (UW3114) strains showed haemolytic activity while none of the recipient strains were haemolytic. The *E. faecalis* transconjugant developed a strong beta-haemolysis after PAI acquisition, while the PAI positive *E. faecium* transconjugant remained non-haemolytic. Since the cytolysin operon can also be plasmid encoded, haemolysis was also tested in transconjugants carrying the *erm*(B) plasmid pLG2 but lacking the PAI (see Table 2) and were confirmed non-haemolytic. Furthermore, sequencing of pLG2 did not reveal the presence of any haemolysin or cytolysin genes (see below). Consequently the acquired haemolytic activity of *E. faecalis* transconjugants could be linked exclusively to the cytolysin operon present within the PAI.

#### Animal experiments

**Figure 8:**
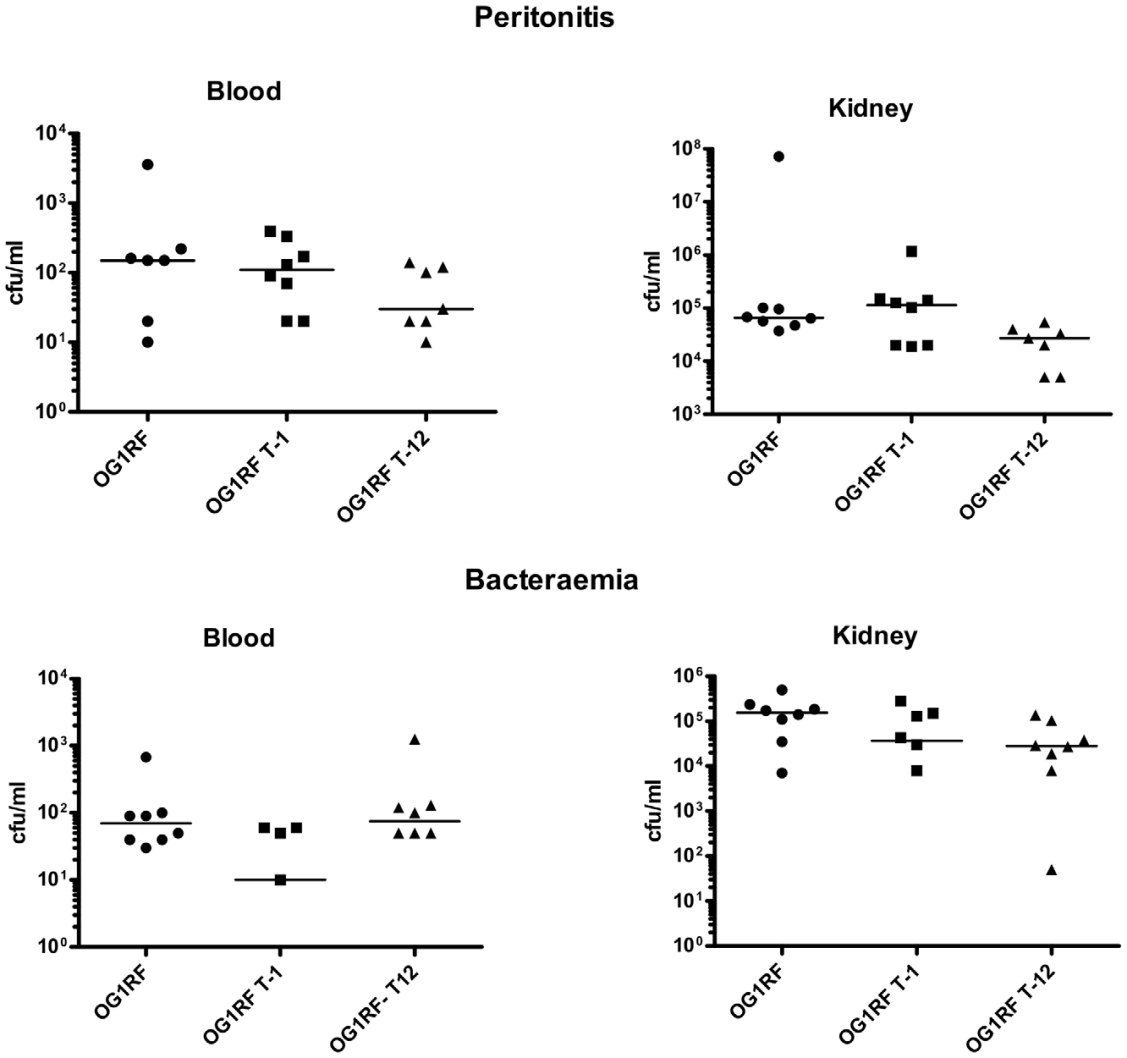
Bacterial counts in the blood (left) and kidneys (right) 24 h after (above) intraperitoneal and (below) intravenous injection of eight female BALB/c mice (6–8-week-old) with *E. faecalis* strains. OG1RF is the recipient strain, OG1RF T-1* (OG1RFxUW3114 T-1) acquired the plasmid pLG2 but lacks the PAI, OG1RF T-12 (OG1RFxUW3114 T-12) is the transconjugant that acquired pLG2 and the *E. faecalis* PAI (See also Table 2). Data represent the individual bacterial counts and the media. No significant differences were observed between the recipient and transconjugants in any of the settings (p>0.50). The inocula used for the bacteraemia model were OG1RF: 8.5×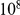, OG1RFxUW3114 T-1: 5.5×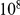, OG1RFxUW3114 T-12: 5.2×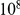. The inocula used for the peritonitis model were OG1RF: 4.4×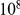, OG1RFxUW3114 T-1: 5.3×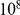, OG1RFxUW3114 T-12: 5.7×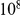.

Mouse bacteraemia and peritonitis models were used to compare the pathogenic potential of *E. faecalis* isogenic strains differing only by presence of the PAI. The bacterial loads in blood and kidneys after 24 h of infection were compared. Kruskal-Wallis analysis indicated that the distributions of the data did not differ significantly, excepting in the bacterial counts of kidneys in the peritonitis model (Peritonitis: blood p = 0.3794, kidney = 0.0327; Bacteraemia: blood p = 0.094, kidney p = 0.088). However Dunn's multiple comparisson post-test indicated no significant differences when comparing the bacterial counts of each strain, meaning that the pathogenic potential of the PAI-positive and PAI negative transconjugant strains was the same (Figure 8).

#### *In vitro* Growth

**Figure 9:**
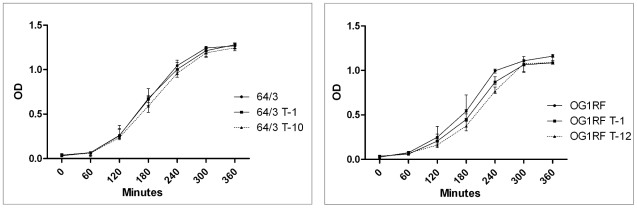
Comparative *in vitro* growth of *E. faecium* (left) and *E. faecalis* (right) recipient and transconjugant strains. 64/3 and OG1RF are the recipient strains. 64/3 T-1 * (64/3xUW3114 T-1) and OG1RF T-1 * (OG1RFxUW3114 T-1) are transconjugants that acquired the *erm*(B) plasmid pLG2 but lack the PAI. Strains 64/3 T-10 (64/3xUW3114 T-10) and OG1RF T-12 (OG1RFxUW3114 T-12) are the transconjugants that carry pLG2 and the *E. faecalis* PAI; comparisson of their growth to that of the corresponding recipient strains indicated no significant differences both in *E. faecium* (p = 0.9909) as in *E. faecalis* (p = 0.9329). Error bars denote standard deviation. Each experiment was repeated three times.

Growth of the recipient strains and the transconjugant strains lacking and carrying the PAI was compared in order to determine differences in growth as a consequence of the altered genome size (ca. 266 kb larger genome in PAI-positive strains). There was no significant difference between the growth of the recipient and the transconjugant strains both in *E. faecium* (p = 0.9909) as in *E. faecalis* (p = 0.9329), as demonstrated by one way ANOVA and Tukey's post test analysis (Figure 9).

### Plasmid sequencing

The plasmid pLG2 was sequenced in order to investigate the presence of gene clusters that might be involved in conjugation and could have supported the horizontal transfer of the *E. faecalis* PAI. The sequences of pLG2 can be found in GeneBank under the accession numbers HQ426650 to HQ426665. 454-sequencing of pLG2 yielded a total of 46 contigs. The largest contig was 33,447 bp long; 17 contigs were larger than 500 bp and covered 53.2 kb. The total number of aligned bases was 62.6 kb, very close to the 66 kb calculated plasmid size. Analysis of the plasmid sequences showed that pLG2 does not correspond entirely to any previously described enterococcal plasmid. Similarities to fragments of different enterococcal plasmids (pAD1, pTEF1, pTEF2, pAM373, pVEF1, pVEF2, pEF1, pIP816, pRE25) were discovered, especially at the replication and mobilization related regions suggesting that pLG2 has a modular structure. Complete replication and mobilization modules including an *oriT* suggest that pLG2 is a conjugative plasmid, theoretically capable of triggering the transfer of the *E. faecalis* PAI. The finding of a pheromone binding protein and the structure of the replication module suggested that pLG2 is a pheromone responding plasmid. Determinants encoding antibiotic resistance to erythromycin, streptomycin and streptothricin were also located within the plasmid sequence (Table 3).

**Table 3:** Annotation of complete identified ORF in the sequencing contigs of pLG2.

Contig	Start	End	Locus Tag	Product
Replication and mobilization				
13	1975	3468	pLG2-0001	similar to plasmid replication protein
13	4068	5033	pLG2-0003	PrgP
13	5005	5280	pLG2-0004	PrgO protein
25	2	373	pLG2-0063	SPOUT methyltransferase superfamily protein
27	3	506	pLG2-0065	Nucleotidyltransferase/DNA polymerase involved in DNA repair
8	1489	2061	pLG2-0012	resolvase
8	2077	2682	pLG2-0013	Filamentation induced by cAMP protein Fic
8	7519	8400	pLG2-0019	DNA nuclease
8	11240	11716	pLG2-0026	single-strand binding protein
8	11856	13190	pLG2-0027	PcfJ
8	24043	24597	pLG2-0038	resolvase
8	30710	32254	pLG2-0047	DNA recombinase, putative
8	32274	32690	pLG2-0048	recombinase
8	32691	33017	pLG2-0049	possible DNA recombinase
8	33021	33188	pLG2-0050	DNA recombinase, putative
17	2	133	pLG2-0058	peptidase
17	126	461	pLG2-0059	peptidase M16
30	3	338	pLG2-0067	cell wall surface anchor family protein
30	447	677	pLG2-0068	cell surface protein
38	14	481	pLG2-0072	pheromone binding protein
31	3	578	pLG2-0069	phage infection protein
Antibiotic resistance				
8	24952	25689	pLG2-0039	erythromycin resistance transferase
8	25790	26155	pLG2-0040	predicted protein
8	26439	27233	pLG2-0041	aminoglycoside 3′-phosphotransferase
8	27326	27481	pLG2-0042	streptothricine-acetyl-transferase
8	27552	27794	pLG2-0043	possible streptothricin acetyltransferase
8	27803	28711	pLG2-0044	streptomycin aminoglycoside 6-adenyltransferase
14	1664	3604	pLG2-0056	tetracycline resistance protein
others				
20	15	503	pLG2-0060	FeS assembly protein sufD
24	10	288	pLG2-0061	regulator of sorbitol operon
24	340	588	pLG2-0062	transcriptional regulator SrlR
26	2	502	pLG2-0064	aspartate kinase
29	2	481	pLG2-0066	GTP-binding protein
43	2	169	pLG2-0073	RinA family transcriptional regulator

Contigs where the ORF is found and exact location within them are given.

We identified a replicase gene, repR-pLG2 (pLG2-0001) that shared 100% identity at the nucleotide level to the replicase genes of *E. faecium* plasmids p5753cB (GQ900487.1), pIP816 (AM932524.1), pVEF1-3 (AM296544.1; AM410096.1; AM931300.1), pEF1 (DQ198088.1), and *E. faecalis* plasmid pRE25 (X92945.2) representing Inc18-type plasmids. Similarity extended to a 5.3 kb region spanning the replicase (contig 13 coordinates 1∶5374). The region spanning the replicase gene was 99% identical to the 3.7 kb replication region of pEF1, which is virtually identical to a homologous region described in pRE25. This region contains a putative replication origin *oriR*, up to five DnaA boxes and several inverted repeats [23]. With regard to the segregation machinery, we identified a putative PrgP-PrgO partitioning system, upstream and inverted to the replicase gene. No ORF that exhibits homology to a relaxase could be identified. The replication and segregation elements identified in pLG2 are listed in Table 3.

All the elements necessary for plasmid mobilization in Gram-positives were identified in pLG2 as can be seen in Table 3. The region between coordinates 2783∶1091 of contig 8 shared 98% similarity at the nucleotide level along 1.6 kb of the 4.8 kb mobilization region of pEF1 (e = 0.0). A resolvase (pLG2-0012) and a “Filamentation induced by cAMP protein-Fic” (pLG2-0013), homologous to pEF1 MobC are encoded in this region.

Within the same contig, an origin of transfer (*oriT*) was identified based on the 99% similarity (expect = 0.0) to the mobilization region of pAD1 where an *oriT* has been previously identified between ORF53 and ORF57 (AF343837) [24]. Other genes identified that are related to plasmid mobilization are listed in Table 3.

Other mobilization related genes of pLG2 include a putative single strand binding protein gene (pLG2-0026) that could act as a coupling protein that binds the DNA for substrate presentation during transfer, two peptidase putative genes (pLG2-0058 and pLG2-0059), which might degrade the bacterial cell wall for the formation of a transfer canal, and two putative resolvase genes (pLG2-0012 and 38) that could be involved in plasmid segregation by resolving plasmid dimers.

## Discussion

The *E. faecalis* PAI is flanked by 10 bp DR, and the first two genes encoded within the PAI are a putative phage-related integrase and an excisionase [1], suggesting that it can excise from and integrate into the chromosome by homologous recombination and can be transferred as a single entity [6]. Our results demonstrate for the first time that the entire *E. faecalis* PAI is capable of precise excision, circularization and horizontal intra- and interspecies transfer. We describe chromosome-to-chromosome transfer and site-specific integration of the *E. faecalis* PAI into the chromosome of *E. faecalis* and *E. faecium*.

The *E. faecalis* strain used as donor, UW3114, had been successfully used in previous studies to demonstrate the intra-species transfer of the *esp* gene into *E. faecalis* [21], however, transfer of further fragments of the PAI could not be investigated in detail because recipient strain *E. faecalis* JH2-2 already possesses parts of the PAI. Coburn *et al.* have described excision of a 27,744 bp internal fragment of the PAI, circularization, integration into a pTEF1-like plasmid and transfer into an *E. faecalis* recipient strain [6]. Recently Manson *et al.* have shown that the *E. faecalis* PAI can transfer into *E. faecalis*, not precisely but as part of larger chromosomal fragments. The horizontal transfer of such regions requires the help of either pTEF1 or pTEF2 pheromone-responsive plasmids and is *recA*-dependent, while the integrase and excisionase genes present in the PAI were not required [7]. However, a role of the integrase and excisionase genes for the precise excision and integration of the PAI cannot be ruled out.

van Shaik *et al.* have recently indicated that some hospital strains of *E. faecium* carry the *esp* gene in a PAI- like structure different from the *E. faecalis* PAI. The *E. faecium esp* PAI can be transferred horizontally among *E. faecium* and contains a 10 kb fragment identical to a gene cluster in the *E. faecalis* PAI that suggests a recent intra-genus transfer or acquisition of the PAIs from a third source [22].

Our analysis of the PAI structure in the donor and the transconjugants demonstrated that it did not undergo any changes during transfer. Compared to the originally described PAI of strain MMH594, the transferred PAI structure contained some deletions and insertions (Figure 1).

The differences observed along the transferred PAI compared to the prototype PAI of strain MMH594 agrees with the previous observation that the gene content of the *E. faecalis* PAI is highly variable and PAI subtypes are widely spread [2], [5], [3]. The cluster-like variability in gene composition and presence of sequences related to mobile genetic elements within the PAI suggest that it has evolved by initial spread of some core elements, and later recombination, acquisition, and loss of gene clusters [2], [3].

The *E. faecalis* PAI is integrated into an non-coding region flanked by an ORF specifying a hypothetical protein with no predictable function and a putative oxidoreductase [1]. The *E. faecalis* PAI of the *E. faecalis* donor strain UW3114 was located into the same region and it also integrated into this region in the recipient strain OG1RF, suggesting a high site-specific integration. In *E. faecium* transconjugant strain 64/3xUW3114 T-10 the *E. faecalis* PAI integrated into a tRNA*lys* gene. This integration site of the *E. faecalis* PAI in *E. faecium* is distinct from the region where the *esp* -encoding PAI of *E. faecium* (AY322150) is integrated in *E. faecium* [25], [22].

The regions of integration of the *E. faecalis* PAI in *E. faecium* and in *E. faecalis* shared 85% similarity at the nucleotide level along a *ca.* 95 bp region. This homology presents the possibility that larger regions act as the recognition sequences for PAI integration, although the 10 bp sequence (attP-like) should be the effective site for homologous recombination, since it resulted in DR (*attL*- and *attR*-like) at both ends after PAI chromosomal integration. It seems evident that the horizontal transfer event reported here resembles that of ICEs.

It is known that due to the short stretches of DNA sequence similarity necessary to initiate recombination events, circular DNA molecules are more likely to be transferred and acquired, making them the most promiscuous recombinogenic states of all DNA [26]. On the other hand circularized intermediates of other PAIs have been seen and associated with their transfer [27] [9]–[10] [17]. These observations prompted us to test for the excision of the PAI from the chromosome and for the formation of circular intermediates.

Besides confirming that the PAI can precisely excise and circularize (only observed in the donor strain UW3114), imprecise excision and circularization were also detected (Fig. 4) apparently due to homology between internal regions of the PAI and flanking chromosomal regions. The circular intermediates of the PAI lacking the integrase and excisionase genes might be unable to integrate/excise in a recipient strain.

The fact that precise excision and circularization could only be detected in the donor strains but not in the reference strain MMH594 might explain why previous attempts to transfer the entire PAI element from this strain have not been successful [6].

Esp protein was detected on the surface of all PAI-positive strains, although FACS analysis revealed a lower *esp* expression in the *E. faecium* transconjugant than in *E. faecalis* (Figure 6). It is possible that the expression of genes encoded within the PAI are affected by regulatory factors inherent to the cell or species-specific. *esp* expression was diminished when cells were grown at 21°C, as previously described for *esp* in *E. faecium* [28]. Temperature-dependent regulation of expression is an ecological feature for regulation of pathogenicity factors also observed in other pathogens that alternate between an environmental reservoir and a mammalian host [28]. Our results show that the temperature-dependent expression of *esp* is also a feature of *E. faecalis* (Figure 6).

The acquisition of the *E. faecalis* PAI increased *in vitro* biofilm formation in *E. faecalis*. However, the acquisition of *E. faecalis* PAI was not sufficient to develop a biofilm forming phenotype in *E. faecium*. The nearly absent biofilm formation of the recipient strain, together with the low Esp on the cell surface, might explain the low biofilm formation in the *E. faecium* PAI-positive strain. Additionally, it is well-known that *E. faecium* has low biofilm forming capacity compared to *E. faecalis* [29], [30], and it has been demonstrated that although *esp* can increase the biofilm forming capacity in *E. faecium* and *E. faecalis* [31],[32],[28],[33], it is neither sufficient nor absolutely necessary for biofilm formation [34], [35].

The cytolysin/haemolysin *cyl* operon encodes a bacterial toxin expressed by some strains of *E. faecalis* which in addition to mediating lysis of erythrocytes, also possesses antibacterial activity toward a broad range of Gram-positive bacteria [36]. The *cyl* operon is either encoded within large, pheromone-responsive plasmids or on the chromosome within the *E. faecalis* PAI [37]. Our PCR results showed that the complete and intact *cyl* operon was present in the transferred *E. faecalis* PAI element. However, a strong (beta) haemolytic activity could only be detected in *E. faecalis* strains. To our knowledge this is the first description of transfer of the *cyl* operon and *cyl* genes to *E. faecium*. Previous studies have reported complete absence of *cyl* genes (n = 271) among *E. faecium* [38] as well as absence of both *cyl* genes and haemolytic activity (n = 21) [39]. Vancanneyt *et al.* have reported beta haemolytic activity in 5 *E. faecium* isolates (n = 78) but no link could be established between haemolytic activity and gene presence [40].

Several bacteriocins have been described in *E. faecium*: enterocin A, enterocin I, enterocin P, enterocin L50A/L50B, and enterocin B [41]; however, none of these bacteriocines and/or the strains producing them possessed haemolytic activity [42], [43], [44], [45], [46].

It remains unclear what prevents the expression or activity of the *E. faecalis* cytolysin/haemolysin in *E. faecium*.

Transfer of the PAI and expression of pathogenicity-associated factors encoded within it prompted us to compare the pathogenicity of the *E. faecalis* isogenic strains carrying and lacking the PAI. Several pathogenicity factors encoded within the *E. faecalis* PAI are demonstrated to contribute to the virulence of *E. faecalis* in different animal models. These include Esp, cytolysin/haemolysin, and aggregation substance (AS), Asc10 [47], [37]. The mouse bacteraemia and peritonitis models did not show a significant difference between isogenic PAI-positive and PAI-negative *E. faecalis* strains (Figure 8). The supposed increase in pathogenicity in PAI-positive transconjugants could be compensated by an impaired growth associated with the enlarged genome size [48], altough the observed diminihsed growth of the *E. faecalis* PAI-positive transconjugant was not significant (Fig. 9). However, enterococci exhibit virulence through mechanisms such as adhesion and biofilm formation, translocation through eukaryotic cell layers, or release of proinflammatory molecules, and the absence of an observable effect in the models used does not exclude an increased pathogenicity of the transconjugants.

pLG2, the *erm*(B) -plasmid transferred along with the PAI, carries all the elements necessary for replication including repR-pLG2 and oriR and segregation locus (PrgP-PrgO). Although a replicase and an origin of replication homologous to pEF1 are present in pLG2, no relaxase could be identified, as has also been described in other pheromone-responsive plasmids (i.e. pAD1, pCF10 and pAM373). Ruiz Barba *et al.* suggest that pEF1 replicates using the *oriR* and *repR* using the 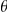-type replication mechanism [23], which could also be true for pLG2 given the homology of its replicase to that of pEF1. However, the mechanism of replication of pheromone-responsive plasmids is still unknown [49]. The PrgP-PrgO partition system of pLG2 (segE locus) constitutes a putative ParAB-like partition system [50] and is putatively involved in replication as it has been observed in pCF10 and pRE25 [51], [52]. The two genes are upstream and inverted with respect to *repR*-pLG2 and constitute another characteristic of pheromone-responsive plasmids. A similar organization of these partition protein genes, normally known as *repBC*, has been previously described in pCF10, pAD1, pPD1, pAM373 and pRE25 [51].

The conjugative transfer of Gram-positive plasmids requires elements that provide contact between the mating cells, the formation of a canal to cross the cell wall, and the transfer of the plasmid DNA. Homologues to all these elements were found in the DNA sequence of pLG2.

One of the mobilization-related regions of pLG2 is homologous to that of bacteriocin-encoding plasmid pEF1 and the other to that of the RepA_N- family plasmid pAD1. Meanwhile, the replicase gene identified was a RepR, which is unrelated to the RepA_N- plasmid family. This suggests a modular composition of pLG2, which is a common trait of enterococcal and other plasmids [53], [54]. Phylogenetic analyses have shown that the *repA* replication gene and *repBC* loci are separable modules that do not need to be closely associated [49].

A recent report of Manson *et al.* describes that pTEF1 and pTEF2 plasmid transfer functions (i.e. *oriT*, relaxase and TraG) were essential for the transfer of chromosomal DNA among *E. faecalis*, and its integration into the recipient's chromosome was recA dependent. BLAST comparisons revealed that pLG2 is different from pTEF1 and pTEF2. However, the mobilization region bearing the *oriT* in pLG2, as well as that of pTEF1 is homologous to the oriT region of pAD1. Despite this congruence, they contain different replication regions, and neither a relaxase nor TraG were detected in pLG2 sequences.

Although not formally proven in the present study by functional knockout experiments, it seems that the pLG2 plasmid resembling conjugative *E. faecalis* pheromone plasmids can support horizontal PAI transfer by providing the conjugative modules necessary for cell-to-cell transfer.

## Materials and Methods

*E. faecalis* strain UW3114 harbouring a variant of the *E. faecalis* PAI which contains the *esp* gene, and an erythromycin resistance plasmid (pLG2), was used as donor. *E. faecalis* strain OG1RF, and *E. faecium* strain 64/3 lacking the *E. faecalis* PAI were used as recipients. Filter mating experiments were done as described elsewhere [55]. Transconjugants were selected on Brain Hearth Infusion (BHI) agar (Difco Labs., Detroit, USA) containing the selective marker for the donor (Erythromycin 10mg/L) and those for the recipient (rifampicin 30mg/L and fusidic acid 20mg/L). Presence of the *esp* gene in the transconjugants, tested by PCR, was indicative of horizontal transfer of the PAI. The strains used are listed in Table 2.

### PCR and long template PCR

The presence of the *esp* gene was tested by PCR using the primers esp1 and esp2 as previously described [21] using Ilustra PuReTaq Ready-To-Go PCR Beads (GE Healthcare Europe GmbH). Primers binding outside the integration site of the PAI PAI164 and PAI167UW3114 or PAI164Efm or PAI167Efm were used to amplify the unoccupied integration site in the *E. faecalis* and *E. faecium* recipient strains. Primers EF2 and ER1 binding inside the PAI (reading outwards the PAI) were used with primers binding outside of the PAI (reading into the PAI) to demonstrate the occupation of the integration site by the PAI in the transconjugants (Table 4) (Figure 4). Presence of the whole *E. faecalis* PAI, based on the sequence of the *E. faecalis* PAI from strain MMH594 [1], was tested by amplification of overlapping regions of *ca.* 10 kb using the Expand long template PCR kit (Roche Biochemicals, Mannheim, Germany) under the conditions recommended by the manufacturer.

**Table 4:** Primers used in this study.

Primer name	Sequence	Reference Sequence	Reference
esp-TIM1	CTTTGATTCTTGGTTGTCGGATAC	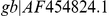	[38]
esp-TIM2	TCCAACTACCACGGTTTGTTTATC	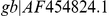	[38]
ermB1	AGCCATGCGTCTGACATCTAT	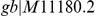	[67]
ermB2	TGCTCATAAGTAACGGTACT	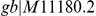	[67]
PAI164	ATGCCATGTTCAGCGAAGTTGCCAATTATC	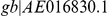	[1]
PAI167	GCTGATTTATTATGGTTCTCAGCAATCGCC	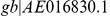	[1]
PAI164Efm	GGCTAAGCCTTCTTGTCTTTTATCGTTAAG	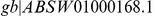	This study
PAI167Efm	TAGTCAATCTAAGCGGGAATGTTGTTTT	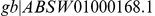	This study
EF2	CCAAAAAGCAACTTTCAACC	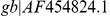	[21]
ER1	ATTCAAGAATGGCTGGGAC	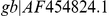	[21]
PAI164nested	TTATACAACGGGGGCATAGC	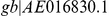	This study
PAI167 UW3114	AAACGTCCTAAGACGCCGACAGAATAC	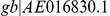	This study
PAI167nested	GATTCGAACCCTAGACCCTCT	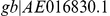	This study
TSP1 5′	GAAGATGGACGGTTGATGAAGCCTC	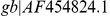	This study
TSP2 5′	GGCTGGGACATGCATCGTATTCG	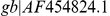	This study
TSP3 5′	CGTGCAGCAGAAGCATTAGAAAACGC	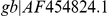	This study
TSP1 3′	GCGGAATTCTGGTATTGAGC	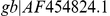	This study
TSP2 3′	TGAACTTGCCAAATCAGTGG	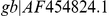	This study
TSP3 3′	CCTACCAATTGCCAAGGAAAT	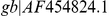	This study

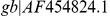
 is E. faecalis PAI, 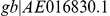 is E. faecalis V583, 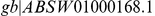 is E. faecium strain U0317.

Regions yielding unexpected size or no PCR amplification product were further analysed by regular PCR of each predicted ORF present within these regions (Primers are listed in Table S2, Table S1). If PCR results suggested that some regions were absent, first regular and then Long template PCRs were performed that would bridge the resulting gap having always confirmed that the binding sites of the primers used were present.

All PCR products were completely or partially sequenced according to the manufacturer recommendations for cycle sequencing of PCR products (Applied Biosystems Germany, Darmstadt, Germany).

Excision and circularization of the *E. faecalis* PAI were tested by two-stage nested PCR. The first PCR was done using the Expand long template PCR kit, with 5min. elongation time. Nested PCR was done using Ilustra PuReTaq Ready-To-Go PCR Beads.

Presence of circular intermediates was investigated with primers TSP2 5′ and TSP2 3′ in the first PCR. For the nested PCR, 1
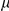
l of PCR product was used for amplification of an inner region thereof using primers TSP3 3′ and TSP3 5′. To investigate the excision of the PAI from the chromosome of *E. faecalis*, primers PAI164 and PAI167 UW3114 were used for the first round of PCR and primers PAI164nested and PAI167nested for the second (Figure 4 and Table 4).

### PFGE and Southern hybridization

Preparation of samples for subsequent macrorestriction analysis was done as previously described [55]. PFGE and analysis of the resulting band patterns were done as described elsewhere [56]. A CHEFF III apparatus (BIO-RAD, Munich, Germany) was used. The sizes of the resolved macrorestriction fragments were predicted according to an external size standard (*Sma*I -digested *Staphylococcus aureus* NCTC8325) and calculated using BioNumerics v. 6.0 software (Applied Maths, Sint-Martens-Latem, Belgium) [57]. Southern hybridization and immunological detection were done using DIG system kits and CDP-Star detection following the manufacturer's recommendations (Roche). Labelled probes were generated using DIG-labelled dUTP (Roche Biochemicals, Mannheim, Germany) using primers esp1 and esp2 for *esp* probes, and primers ermB1 and ermB2 for *erm*(B) probes.

#### I-*Ceu*I macrorestriction

I-*Ceu*I cuts inside ribosomal operons and thus linearizes chromosomal DNA. Chromosomal location of *esp* was analysed by Southern hybridization of I-*Ceu*I digested genomic DNA resolved in PFGE as previously described [58].

#### S1-nuclease macrorestriction

Plasmid content of the strains and possible plasmid localization of *esp* and *erm*(B) were analysed by S1-nuclease treatment and subsequent Southern hybridization analysis [59], [60]. Briefly, genomic DNA was digested with 14U of S1 nuclease (Takara, Bio Inc., Shiga, Japan)for 15 minutes at 37°C. DNA was separated in PFGE using ramped pulse field times as follows: 5-35 seconds for 22 hours at 14°C. This method is based on the specific digestion of single-stranded DNA or single-stranded regions of double-stranded DNA allowing the linearization of plasmids and resolution in PFGE as detectable bands on a faint genomic background [59].

### Genomic walking

The integration site of the PAI in *E. faecium* was determined using a DNA Walking *Speedup* Premix kit (Seegene, Seoul, Korea). Specific primers: TSP1 5′, TSP2 5′ and TSP3 5′, tagging the 5′end of the PAI and TSP1 3′, TSP2 3′, TSP3 3′ tagging the 3′ end of *E. faecalis* PAI (Table 4 and Figure 4) were designed and used according to the recommendations of the manufacturer.

### Phenotypic changes by PAI acquisition

Differences by acquisition of the *E. faecalis* PAI were assessed on the *E. faecium* and *E. faecalis* isogenic set of strains: plasmid-free recipients lacking the PAI, transconjugants that acquired a conjugative *erm*(B) -plasmid but lack the PAI (64/3xUW3114 T-1 and OG1RFxUW3114 T-1) and transconjugants bearing both the *erm*(B) -plasmid and the PAI (64/3xUW3114 T-10 and OG1RFxUW3114 T-12) (Table 2).

#### Cell surface expression of *esp*

Transmission electron microscopy (TEM) and Fluorescence activated cell sorting (FACS) were used for detection of Esp on the bacterial surface as previously described [28], [61].

Esp expressed on the cell surface was visualized after immunologic labeling with gold particles. Briefly 200-mesh Formvar-carbon-coated copper grids were floated on drops of bacterial suspensions (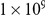 CFU/ml) for 10 min.. Grids were washed and blocked with 1% bovine serum albumin in PBS (PBSb). Esp was labelled by floating the grids for 1 hour on drops containing a 1/100 dilution of anti-Esp rabbit immune serum in PBSb. Antibodies were labelled with protein A-Gold (10 nm) in PBS. Grids were washed four times, fixed, and bacteria were stained with 1.8% methylcellulose (25 centipoises -Sigma-Aldrich) and 0.4% uranyl acetate (pH 4) and subsequently air dried for 10 min.. Grids were examined using a Jeol 1010 transmission electron microscope (Jeol-Europe, Amsterdam, The Netherlands) [28].

For FACS aanalysis 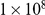 bacterial cells were incubated for 30 minutes in 50 ml RPMI 1640 supplemented with 0.05% human serum albumin (HAS) containing 1∶100 anti Esp-rabbit immune serum. After washing, cells were incubated for additional 30 min. in 50
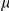
l RPMI 1640-HAS containing 1∶50 goat anti-rabbit flourescein isothiocyanate (Sigma-Aldrich, Saint Louis, USA). Bacteria were washed and resuspended in 300 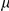
l RPMI 1640-HSA before analyses in a FACSCalibur (BD, Alphen aan den Rijn, The Netherlands). All measurements were performed with the same apparatus using the same parameters. The data were normalized for bacterial size, and experiments were performed in triplicate. The mean fluorescence (channel 1) was used as a measure for cell surface-associated Esp. Samples incubated without anti-Esp rabbit immune serum were used as conjugate controls [28].

#### Biofilm formation

*In vitro* biofilm formation was evaluated on polystyrene microtiter plates following the methodology described previously [33] with some modifications. Briefly, bacteria were grown on blood agar plates and resuspended in Tryptic Soy broth supplemented with 0.25% glucose to a concentration of 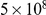 CFU/ml. 100
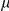
l bacterial suspension was added in triplicate per well using Flat bottomed 96-well polystyrene microtiter plates (Greiner Bio-one, Germany and Corning Inc., NY, USA). After incubation at 37°C for 24 hours, bacterial suspensions were removed and the wells were washed three times with 200
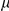
l of PBS. The plates were dried for 1 h at 60°C and stained with 100
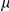
l of 0.2% Gram's crystal violet solution (Merk, Darmstadt, Germany) for 15 minutes at room temperature. The dye was removed and the wells washed again with 200
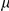
l PBS three times. The plates were dried for 10 min at 60°C. The dye was resuspended for 30 minutes by adding 100 ml of ethanol 100% into each well. Absorbance at 595 nm was measured with a Sunrise ELISA reader (Tecan Group,Maennedorf, Switzerland). The experiment was repeated 3 times.

#### Cytolysin/Haemolysin

The haemolytic activity of the strains was detected using Blood agar plates, as elsewhere described [62]. Strains were streaked onto agar plates containing BHI agar with 5% (w/v) defibrinated human erythrocytes, 1% (w/v) glucose and 0.03% (w/v) L-arginine (Sigma-Aldrich, Saint Louis, USA).

#### Animal experiments

The animal welfare committees of the university of Freiburg (RegierungsprÃ¤sidium Freiburg Az 35/9185.81/G-07/15) approved all animal experiments. The pathogenic potential and *in vivo* survival of strains was assessed in a mouse bacteraemia model and a peritonitis model as described previously [63], [64]. Briefly, eight 6-8-week-old female BALB/c mice were challenged by intravenous (via tail vein) or intraperitoneal injection of 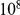 CFU of each strain. 24 h after infection, the mice were sacrificed, exsanguinated by cardiac puncture and kidneys were sterile harvested. Bacteria were enumerated in blood and homogenized kidneys by serial dilutions and plating.

#### Growth

*In vitro* differential growth of the strains was tested by measuring absorbance (OD-600 nm). 50
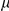
l aliquots of inoculum (*ca.* 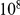 cells/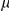
l) were added to 2,45 ml of BHI broth and grown in agitation at 37°C. OD-600 was measured every 60 minutes until the stationary phase was reached. Each experiment was done in triplicate.

#### Statistical analysis

Data from *in vitro* growth and biofilm forming capacity of the recipient strains Vs. their corresponding PAI positive strains were compared using one way ANOVA followed by Tukey's multiple comparison test. Results from FACS were analyzed using two way ANOVA to compare statistical significance of temperature on *esp* expression; One way ANOVA and Tukey's post test were applied to compare the *esp* expression of the different strains at 37°C. Data from animal experiments were analyzed applying Kruskal-Wallis test followed by multiple comparissons using Dunn's post-test. All statistical analysis were done using GraphPad Prism5 software.

### Plasmid DNA isolation

Plasmids were isolated using a phenol/chloroform-based extraction method as previously described [65].

The isolated plasmid preparations were analysed by agarose gel electrophoresis for visual inspection. The DNA concentration was determined using Quant-iT® PicoGreen® dsDNA Quantitation Reagent following the instructions of the manufacturer (Invitrogen®- Molecular Probes Inc., Paisley, UK).

### Plasmid sequencing

Isolated plasmid DNA (*ca.* 5 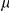
g) from PAI-positive *E. faecalis* transconjugant strain OG1RFxUW3114 T-12 was subjected to 454 sequencing at GATC Biotech (Konstanz, Germany). The plasmid sequencing and the single read library preparation were done according to standard procedures for sequencing on the Genome Sequencing (GS) FLX system (Roche). The pre-assembled contigs were analysed using DNA-Star® software SeqMan Engine and DS Gene software packages. Coding sequence (CDS) prediction and annotation were done with Kodon® (Applied Maths, Texas, U.S.A.) and the help of BLAST programs (http://blast.ncbi.nlm.nih.gov/Blast.cgi) [66].

## Supporting Information

Table S1
**Primers used for investigation of** ***E. faecalis*** **PAI presence by long template- PCR.** PAI regions were chosen arbitrarily as *ca.* 10 Kb overlapping amplicons based on the *E. faecalis* PAI structure of Strain MMH594 (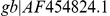). C, binding site is in the chromosome of *E. faecalis*.(PDF)

Table S2 **Primers used for investigation of** ***E. faecalis*** **PAI presence by regular PCR.**
(PDF)

## References

[1] Shankar N, Baghdayan AS, Gilmore MS (2002). Modulation of virulence within a pathogenicity island in vancomycin-resistant Enterococcus faecalis.. Nature.

[2] McBride SM, Coburn PS, Baghdayan AS, Willems RJL, Grande MJ (2009). Genetic Variation and Evolution of the Pathogenicity Island of Enterococcus faecalis.. J Bacteriol.

[3] McBride SM, Fischetti VA, Leblanc DJ, Moellering RCJ, Gilmore MS (2007). Genetic diversity among Enterococcus faecalis.. PLoSONE.

[4] Aakra A, Nyquist OL, Snipen L, Reiersen TS, Nes IF (2007). Survey of genomic diversity among Enterococcus faecalis strains by microarray-based comparative genomic hybridization.. Appl Environ Microb.

[5] Shankar N, Baghdayan AS, Willems R, Hammerum AM, Jensen LB (2006). Presence of pathogenicity island genes in Enterococcus faecalis isolates from pigs in Denmark.. J Clin Microbiol.

[6] Coburn PS, Baghdayan AS, Dolan GT, Shankar N (2007). Horizontal transfer of virulence genes encoded on the Enterococcus faecalis pathogenicity island.. Mol Microbiol.

[7] Manson JM, Hancock LE, Gilmore MS (2010). Mechanism of chromosomal transfer of Enterococcus faecalis pathogenicity island, capsule, antimicrobial resistance, and other traits..

[8] Burrus V, Waldor MK (2004). Shaping bacterial genomes with integrative and conjugative elements.. Res Microbiol.

[9] Murphy RA, Boyd EF (2008). Three pathogenicity islands of Vibrio cholerae can excise from the chromosome and form circular intermediates.. J Bacteriol.

[10] Rajanna C, Wang J, Zhang D, Xu Z, Ali A (2003). The vibrio pathogenicity island of epidemic Vibrio cholerae forms precise extrachromosomal circular excision products.. J Bacteriol.

[11] Doublet B, Boyd D, Mulvey MR, Cloeckaert A (2005). The Salmonella genomic island 1 is an integrative mobilizable element.. Mol Microbiol.

[12] Sakellaris H, Luck SN, Al-Hasani K, Rajakumar K, Turner SA (2004). Regulated site-speci_crecombination of the she pathogenicity island of Shigella exneri.. Mol Microbiol.

[13] Middendorf B, Hochhut B, Leipold K, Dobrindt U, Blum-Oehler G (2004). Instability of pathogenicity islands in uropathogenic Escherichia coli 536.. J Bacteriol.

[14] Hochhut B, Wilde C, Balling G, Middendorf B, Dobrindt U (2006). Role of pathogenicity island-associated integrases in the genome plasticity of uropathogenic Escherichia coli strain 536.. Mol Microbiol.

[15] O'Shea YA, Boyd EF (2002). Mobilization of the Vibrio pathogenicity island between Vibrio cholerae isolates mediated by CP-T1 generalized transduction.. FEMS Microbiol Lett.

[16] Ruzin A, Lindsay J, Novick RP (2001). Molecular genetics of SaPI1–a mobile pathogenicity island in Staphylococcus aureus.. Mol Microbiol.

[17] Ubeda C, Tormo MA, Cucarella C, Trotonda P, Foster TJ (2003). Sip, an integrase protein with excision, circularization and integration activities, defines a new family of mobile Staphylococcus aureus pathogenicity islands.. Mol Microbiol.

[18] Muniesa M, Schembri MA, Hauf N, Chakraborty T (2006). Active genetic elements present in the locus of enterocyte effacement in Escherichia coli O26 and their role in mobility.. Infect Immun.

[19] Schubert S, Darlu P, Clermont O, Wieser A, Magistro G (2009). Role of intraspecies recombination in the spread of pathogenicity islands within the Escherichia coli species.. PLoSPathog.

[20] Lesic B, Carniel E (2005). Horizontal transfer of the high-pathogenicity island of Yersinia pseudotuberculosis.. J Bacteriol.

[21] Oancea C, Klare I, Witte W, Werner G (2004). Conjugative transfer of the virulence gene, esp, among isolates of Enterococcus faecium and Enterococcus faecalis.. J Antimicrob Chemoth.

[22] van Schaik W, Top J, Riley DR, Boekhorst J, Vrijenhoek JEPV (2010). Pyrosequencing-based comparative genome analysis of the nosocomial pathogen Enterococcus faecium and identification of a large transferable pathogenicity island.. BMC Genomics.

[23] Ruiz-Barba JL, Floriano B, Maldonado-Barragan A, Jimenez-Diaz R (2007). Molecular analysis of the 21-kb bacteriocin-encoding plasmid pEF1 from Enterococcus faecium 6T1a.. Plasmid.

[24] Francia MV, Haas W, Wirth R, Samberger E, Muscholl-Silberhorn A (2001). Completion of the Nucleotide Sequence of the Enterococcus faecalis Conjugative Virulence Plasmid pAD1 and Identification of a Second Transfer Origin.. Plasmid.

[25] Leavis H, Top J, Shankar N, Borgen K, Bonten M (2004). A novel putative enterococcal pathogenicity island linked to the esp virulence gene of Enterococcus faecium and associated with epidemicity.. J Bacteriol.

[26] Thomas CM, Nielsen KM (2005). Mechanisms of, and barriers to, horizontal gene transfer between bacteria.. NatRevMicrobiol.

[27] Lesic B, Bach S, Ghigo JM, Dobrindt U, Hacker J (2004). Excision of the high-pathogenicity island of Yersinia pseudotuberculosis requires the combined actions of its cognate integrase and Hef, a new recombination directionality factor.. Mol Microbiol.

[28] van Wamel WJ, Hendrickx AP, Bonten MJ, Top J, Posthuma G (2007). Growth condition- dependent esp expression by Enterococcus faecium affects initial adherence and biofilm formation.. Infect Immun.

[29] Mohamed JA, Huang DB (2007). Biofilm formation by enterococci.. J Med Microbiol.

[30] Sandoe JA, Witherden IR, Cove JH, Heritage J, Wilcox MH (2003). Correlation between enterococcal biofilm formation in vitro and medical-device-related infection potential in vivo.. J Med Microbiol.

[31] Tendolkar PM, Baghdayan AS, Gilmore MS, Shankar N (2004). Enterococcal surface protein, Esp, enhances biofilm formation by Enterococcus faecalis.. Infect Immun.

[32] Toledo-Arana A, Valle J, Solano C, Arrizubieta MJ, Cucarella C (2001). The enterococcal surface protein, Esp, is involved in Enterococcus faecalis biofilm formation.. Appl Environ Microb.

[33] Heikens E, Bonten MJM, Willems RJL (2007). Enterococcal surface protein Esp is important for biofim formation of Enterococcus faecium E1162.. J Bacteriol.

[34] Kristich CJ, Li YH, Cvitkovitch DG, Dunny GM (2004). Esp-independent bio_lm formation by Enterococcus faecalis.. J Bacteriol.

[35] Rosa R, Creti R, Venditti M, D'Amelio R, Arciola CR (2006). Relationship between biofilm formation, the enterococcal surface protein (Esp) and gelatinase in clinical isolates of Enterococcus faecalis and Enterococcus faecium.. FEMS Microbiol Lett.

[36] Gilmore MS, Segarra RA, Booth MC, Bogie CP, Hall LR (1994). Genetic structure of the Enterococcus faecalis plasmid pAD1-encoded cytolytic toxin system and its relationship to lantibiotic determinants.. J Bacteriol.

[37] Gilmore MS, Coburn PS, Nallapareddy SR, Murray BE (2002). The enterococci: Pathogenesis, Molecular Biology and Antibiotic Resistance, Washington, D.C.: ASM Press, volume 1, chapter Enterococcal virulence.

[38] Vankerckhoven V, Van Autgaerden T, Vael C, Lammens C, Chapelle S (2004). Development of a multiplex PCR for the detection of asa1, gelE, cylA, esp, and hyl genes in enterococci and survey for virulence determinants among European hospital isolates of Enterococcus faecium.. J Clin Microbiol.

[39] Hallgren A, Claesson C, Saeedi B, Monstein HJ, Hanberger H (2009). Molecular detection of aggregation substance, enterococcal surface protein, and cytolysin genes and in vitro adhesion to urinary catheters of Enterococcus faecalis and E. faecium of clinical origin.. Int J Med Microbiol.

[40] Vancanneyt M, Lombardi A, Andrighetto C, Knijff E, Torriani S (2002). Intraspecies genomic groups in Enterococcus faecium and their correlation with origin and pathogenicity.. Appl Environ Microb.

[41] Del CR, Tenorio C, Jimenez-Diaz R, Rubio C, Gomez-Lus R (2001). Bacteriocin production in vancomycin-resistant and vancomycin-susceptible Enterococcus isolates of different origins.. Antimicrob Agents Ch.

[42] Aymerich T, Holo H, Havarstein LS, Hugas M, Garriga M (1996). Biochemical and genetic characterization of enterocin A from Enterococcus faecium, a new antilisterial bacteriocin in the pediocin family of bacteriocins.. Appl Environ Microb.

[43] Floriano B, Ruiz-Barba JL, Jimenez-Diaz R (1998). Purification and genetic characterization of enterocin I from Enterococcus faecium 6T1a, a novel antilisterial plasmid-encoded bacteriocin which does not belong to the pediocin family of bacteriocins.. Appl Environ Microb.

[44] Cintas LM, Casaus P, Havarstein LS, Hernandez PE, Nes IF (1997). Biochemical and genetic characterization of enterocin P, a novel sec-dependent bacteriocin from Enterococcus faecium P13 with a broad antimicrobial spectrum.. Appl Environ Microb.

[45] Cintas LM, Casaus P, Holo H, Hernandez PE, Nes IF (1998). Enterocins L50A and L50B, two novel bacteriocins from Enterococcus faecium L50, are related to staphylococcal hemolysins.. J Bacteriol.

[46] Casaus P, Nilsen T, Cintas LM, Nes IF, Hernandez PE (1997). Enterocin B, a new bacteriocin from Enterococcus faecium T136 which can act synergistically with enterocin A.. Microbiology.

[47] Sava IG, Heikens E, Huebner J (2010). Pathogenesis and immunity in enterococcal infections.. Clin Microbiol Infect.

[48] Trevors JT (1996). Genome size in bacteria.. Antonie Van Leeuwenhoek.

[49] Weaver KE, Rice LB, Churchward G (2002). The enterococci: Pathogenesis, Molecular Biology and Antibiotic Resistance, Washington, DC: ASM press, volume 1, chapter Plasmids and Transposons.

[50] Lioy VS, Pratto F, de la Hoz AB, Ayora S, Alonso JC (2010). Plasmid pSM19035, a model to study stable maintenance in Firmicutes.. Plasmid.

[51] Hirt H, Manias DA, Bryan EM, Klein JR, Marklund JK (2005). Characterization of the pheromone response of the Enterococcus faecalis conjugative plasmid pCF10: complete sequence and comparative analysis of the transcriptional and phenotypic responses of pCF10-containing cells to pheromone induction.. J Bacteriol.

[52] Schwarz S, Kehrenberg C, Walsh TR (2001). Use of antimicrobial agents in veterinary medicine and food animal production.. Int J Antimicrob Agents.

[53] Weaver KE, Kwong SM, Firth N, Francia MV (2009). The RepA N replicons of Gram-positive bacteria: a family of broadly distributed but narrow host range plasmids.. Plasmid.

[54] Laverde-Gomez JA, Francia MV, Weaver DM, Freitas AR, Coque TM (2010). A multiresistance megaplasmid bearing a hyl-Efm genomic island in hospital Enterococcus faecium isolates..

[55] Werner G, Willems RJ, Hildebrandt B, Klare I, Witte W (2003). Inuence of transferable genetic determinants on the outcome of typing methods commonly used for Enterococcus faecium.. J Clin Microbiol.

[56] Werner G, Fleige C, Ewert B, Laverde-Gomez JA, Klare I (2010). High-level ciprooxacin resistance among hospital-adapted Enterococcus faecium (CC17).. Int J Antimicrob Agents.

[57] Murchan S, Kaufmann ME, Deplano A, de RR, Struelens M (2003). Harmonization of pulsed-field gel electrophoresis protocols for epidemiological typing of strains of methicillin-resistant Staphylococcus aureus: a single approach developed by consensus in 10 European laboratories and its application for tracing the spread of related strains.. J Clin Microbiol.

[58] Novais C, Coque TM, Sousa JC, Baquero F, Peixe L (2004). Local genetic patterns within a vancomycin-resistant Enterococcus faecalis clone isolated in three hospitals in Portugal.. Antimicrob Agents Ch.

[59] Barton BM, Harding GP, Zuccarelli AJ (1995). A general method for detecting and sizing large plasmids.. Anal Biochem.

[60] Freitas AR, Tedim-Pedrosa A, Novais C, Peixe L, Baquero F (2009). Diversity of hyl plasmids among international CC17 Enterococcus faecium strains (1992-2009)..

[61] Hendrickx APA, Van Wamel WJB, Posthuma G, Bonten MJM, Willems RJL (2007). Five genes encoding surface exposed LPXTG proteins are enriched in hospital-adapted Enterococcus faecium Clonal Complex-17 isolates.. J Bacteriol.

[62] Solheim M, Aakra A, Snipen LG, Brede DA, Nes IF (2009). Comparative genomics of Enterococcus faecalis from healthy Norwegian infants.. BMCGenomics.

[63] Hufnagel M, Koch S, Creti R, Baldassarri L, Huebner J (2004). A putative sugar-binding transcrip- tional regulator in a novel gene locus in Enterococcus faecalis contributes to production of biofilm and prolonged bacteremia in mice.. J Infect Dis.

[64] Theilacker C, Sanchez-Carballo P, Toma I, Fabretti F, Sava I (2009). Glycolipids are involved in biofilm accumulation and prolonged bacteraemia in Enterococcus faecalis.. Mol Microbiol.

[65] Werner G, Klare I, Konstabel C, Witte W (2007). The current MLVA typing scheme for Enterococcus faecium does not discriminate enough to resolve epidemic-virulent, hospital-adapted clonal types.. BMC Microbiology.

[66] Zhang Z, Schwartz S, Wagner L, Miller W (2000). A greedy algorithm for aligning DNA sequences.. J Comput Biol.

[67] Shaw JH, Clewell DB (1985). Complete nucleotide sequence of macrolide-lincosamide-streptogramin B-resistance transposon Tn917 in Streptococcus faecalis.. J Bacteriol.

