# Supplementary material for: Intra- and Interspecies Genomic Transfer of the Enterococcus faecalis Pathogenicity Island
Source: PLoS One. 2011 Apr 29;6(4):e16720. doi: 10.1371/journal.pone.0016720 (PMC3084688; doi:10.1371/journal.pone.0016720)
Supplement: Table S1 — Primers used for investigation of E. faecalis PAI presence by long template- PCR. PAI regions were chosen arbitrarily as ca. 10 Kb overlapping amplicons based on the E. faecalis PAI structure of Strain MMH594 (). C, binding site is in the chromosome of E. faecalis. (PDF) [file pone.0016720.s001.pdf]

Table S. 1. Primers used for investigation of *E. faecalis* PAI presence by long template-PCR.

PAI region	Location in $gb|AF454824.1|$	Primer name	Primer sequence	Amplicon (bp)
1	C:427	PAI164	ATGCCATGTTCAGCGAAGTTGCCAATTATC	1548
		1 PAI R	GGAAGATGGACGGTTGATGAAGCCTCAATATG	
2a	240:9913	2a PAI F	CAGTTGTCGAATACGATGCATGTCCCAGCC	9674
		2a PAI R	AAACCAAAGGAACCGAAACGGAAAAACTTAGCATGG	
2b	9869:15934	2b PAI F	TTTAACCAGCCATGCTAAGTTTTTCCGTTTCGGTTC	6066
		2b PAI R	TTTGAAATAATCTCCAACTTTTCCCCCGTTCCACAC	
2c	14033:21999	2c PAI F	AACCATAAAAAGGAACGGAGGGAGCACAACAAAAGG	7697
		2c PAI R	ACTTGCAGTGTGACTGTCTGTCGTAACTTCACC	
3a	21566:32079	3a PAI F	CTCGTCCGTAACGATCTGTTTTATCGCCCTTATC	11645
		3a PAI R	TCAAGTCCGTACAACAGGCACTTTCTTTATCAAGC	
3b	31320:42730	3b PAI F	GAAGGCCGTTGCCAATTTTGCATTAGCTTGC	11411
		3b PAI R	TCCTAAGCCTATGGTAAAACATGCTGGAGTTGTCTC	
4a	42395:53473	4a PAI F	CAAGGTAGTGGAGATGTTCAGGCTGAGACAACAC	11079
		4a PAI R	CGGATGTTACTTCTGCTGGACTTAAAACAATCCC	
4b	53440:65000	4b PAI F	GGGATTGTTTTAAGTCCAGCAGAAGTAACATCCG	11561
		4b PAI R	ACGCCAAGCACAAGGGATAAAGATTGCGAAAG	
5a	64944:75572	5a PAI F	GGACGACCTTTATAGACGCCGTTTGCTTTCG	10629
		5a PAI R	AGTCCCCTTTTTCTGCCATGACACCAGTTAAAATC	
5b	74158:85588	5b PAI F	GCTGTGGTCAAGATAGATGGGAAAGAGATTGAGCG	11431
		5b PAI R	GGATCTGAACCGTCTTGTGTCATAGTGTGCCAG	
6a	85547:94661	6a PAI F	TGTAGCATACTGGCACACTATGACACAAGACGG	9115
		6a PAI R	CGTGCCCCTAATTACCATAGAGATAGTCGCGTTG	
6b	93984:102421	6b PAI F	TGGTAAACGCTGCTCCTGAAATGAAGAGTTTGAC	8432
		6b PAI R	AGGTTTGATACGCAACTACCTTTCCCAACTGACG	
7a	101954:113046	7a PAI F	TTTTGGGACAGGAACGCTATCAGTTAACGATTGC	10821
		7a PAI R	CCTGCGGTCAAGCACAGTTGCCTTATCTTAG	
7b	113008:126865	7b PAI F	ATTAAAGTCAAAAGAGACTGTTACTTGTGCGCCCTG	13858
		7b PAI R	TCAGCAAACTAAGATAAGGCAACTGTGCTTGACC	
8a	125344:136351	8a PAI F	TGCTTTAGTGGGTCGTACTAACGGAACAATAG	11008
		8a PAI R	CAAACAACACGTCGTCGATCTTTACCTTG	
8b	135337:146384	8b PAI F	CACCAATGCACATAATCAAACAATTCTAGGCGTAG	11048
		8b PAI R	GTGGACAAGCACAGTCACAATTAGAAGCAATG	
9a	146272:C	9 PAI F	CATCATTTCTTCAGCAAATTGGTTGGCACGC	8298
		PAI167R	ATGTTGGTTGAAAGTTGCTTTTTGGCAAAC	

PAI regions were chosen arbitrarily as *ca.* 10 Kb overlapping amplicons based on the *E. faecalis* PAI structure of Strain MMH594 ($gb|AF454824.1|$). C, binding site is in the chromosome of *E. faecalis.*
