# Supplementary material for: Intra- and Interspecies Genomic Transfer of the Enterococcus faecalis Pathogenicity Island
Source: PLoS One. 2011 Apr 29;6(4):e16720. doi: 10.1371/journal.pone.0016720 (PMC3084688; doi:10.1371/journal.pone.0016720)
Supplement: Table S2 — Primers used for investigation of E. faecalis PAI presence by regular PCR. (PDF) [file pone.0016720.s002.pdf]

Table S. 2. Primers used for investigation of *E. faecalis* PAI presence by regular PCR.

Primer name	Primer sequence	Amplicon (bp)
PAIefs-11F	AGAATTGTCTTTGGGCTTCTC	378
PAIefs-11R	GCCGAATGTTGCTAAAGTTAC	
PAIefs-12F	GGAAAATGTTTAAAAGCCCCC	486
PAIefs-12R	GGCAACGTGAGAGTAACAG	
PAIefs-21F	GCCATTAAAAAGTAAAGAGCCG	500
PAIefs-21R	CTTGATATGCGCGATAAAGTTG	
PAIefs-55F	AGGATTGCTTACGGTTGATGG	357
PAIefs-55R	TTTGCTTTCTCTGTTTGCTCAC	
PAIefs-58F	GGTGTTGTTCCTGTGGTTAG	536
PAIefs-58R	TTAGGAGTCGTCCCTGTTATC	
PAIefs-65F	TTTTCCAGCTAAAGAACCACC	446
PAIefs-65R	GTAAATACCACGATGAAGTTGC	
PAIefs-70F	CTTGGGATAGTGAACAAGATGG	377
PAIefs-70R	CAAGGACTGCGCTATGAAG	
PAIefs-84F	GCCGATCCTACATATACAAACG	417
PAIefs-84R	TCCAATAGTTGATCATACTGCG	
PAIefs-87F	GTTATTGGCAGTGCCGTTAG	433
PAIefs-87R	GACTTGTGAAAGTCCTCGAAG	
PAIefs-89F	AAGTATTCTAGGCAGTTTGCG	403
PAIefs-89R	GCCACGTTCTTTAGAAATTCG	
PAIefs-91F	TTGGGACAGGAACGCTATC	487
PAIefs-91R	GGCCCCATACTTTTGTCATC	
PAIefs-92F	GAAACAGGTAGACGCTAACG	521
PAIefs-92R	AACGTACTTCTTTTCCCGC	
PAIefs-95F	GGAAACTGGAGGAAATGCTTG	508
PAIefs-95R	CGGTCTATCGTCCACACTAC	
PAIefs-99F	AATTTGGACTTCCAAGCGAC	530
PAIefs-99R	ACTATTTCATTCCCCAGGGC	
PAIefs-102F	GCGCATTTAAGAGTGCAAATTG	582
PAIefs-102R	TGCGGCTAGTAAGTAACCAAC	
PAIefs-108F	AATGCAAACCAGCTCTTAGAC	409
PAIefs-108R	GGTCGATGGTCAATTCATCAG	
PAIefs-111F	GTGACGACAGAAGAACAACTAC	585
PAIefs-111R	TCTCTTGAAAGGAACTGCCC	
PAIefs-115F	CCACCAATGCACATAATCAAAC	471
PAIefs-115R	GTAATGAAGCGGAGACAACG	
PAIefs-122F	TGTTTCAGGTTCAAGTTCAGC	505
PAIefs-122R	TCAACCTCAATGGAAAATGTGG	
PAIefs-128F	ACCAGAAAAACTGAAAGAGACG	548
PAIefs-128R	GACTGGAAGCATTTTGGGC	
PAIefs-13F	AAAGAGCAGCAAGTCCTAAAG	293
PAIefs-13R	TGAGAAACGGTCACAACAC	
PAIefs-14F	AAGGCAATGCTCAATCAGG	251
PAIefs-14R	CCTACGTATCTTCTTTTCGGTG	
PAIefs-15F	TATGAACGCCCAGCGATTC	202
PAIefs-15R	GGGGGACTTTACTTTCTACGAC	
PAIefs-16F	TTTAGCTGCTGGATTGCG	229
PAIefs-16R	CGTTGGTCTATCAAGCAACTTC	
PAIefs-17F	GGTGATTTTAGGACTGGTATCC	199
PAIefs-17R	TCGCCAACCTTTTATTGGG	
PAIefs-18F	TTTGCGCCAAACGGCATTC	181
PAIefs-18R	AAGAAAAGAGCGTCCACACAAG	
PAIefs-19F	AGATTACACCGAGGATGTAGC	174
PAIefs-19R	TCTTCGCTCTCACAATCAAAAG	
PAIefs-20F	TCAACTGTGTTATTTGGCGG	218
PAIefs-20R	CGTAGCTTTACCTGTAGCCTG	
PAIefs-57F	ACGTCAAACCGTATCCAAAG	179
PAIefs-57R	AGCCGTCCGTAATAAAAAGC	
PAIefs-59F	GCAGAAAAAGGGAAGAAATTACAAC	289
PAIefs-59R	CTGGAAAAATACCGAATCTTAGTTG	
PAIefs-60F	GCTTGACCAGATGACTAATGC	180
PAIefs-60R	GCAACGAACTCATCAACAAATG	
PAIefs-61F	GGATTTCCGATTGCTTCAGC	198
PAIefs-61R	AGCGACTATTACTCATCGACC	
PAIefs-62F	GAAGCCATTGACCGATTAGG	185
PAIefs-62R	TGTTCCGCAATCATTGCC	
PAIefs-66F	TCAAGCTGGTGTGGATTTTG	198
PAIefs-66R	CCACCAACGACAATTCCTG	
PAIefs-67F	GCAACTAGCTGATCCAAACC	181
PAIefs-67R	AAATGAAGTCAAGCCACCAC	
PAIefs-68F	TTGACTGGACGAATTGAAGC	222
PAIefs-68R	GACAGATGGATCTTTTCCTGC	
PAIefs-69F	GCCATACTTCCGTCAATAACG	163
PAIefs-69R	TGTCACTACCATTAATCACTGC	
PAIefs-72F	TGAAAATGGCACTCCAACAG	226
PAIefs-72R	CCGTACATCATAGGCACAATC	
PAIefs-73F	GAGTACTGGATTATTCAGGCG	212
PAIefs-73R	TTGAATGGCTTCTTCACCG	
PAIefs-74F	GGGATAAGCAGACGATTATCGG	155
PAIefs-74R	TCTTGTGCGTGGTGCTAAC	
PAIefs-76F	TGCCATAATGGGTATGAGGAC	212
PAIefs-76R	CATGCTGACCTTTAACGGTAG	
PAIefs-78F	GGTTCACAAAGCTGAAGCAG	198
PAIefs-78R	CCTCCAGCAACAGTTAATCC	
PAIefs-79F	AGAGAATGCTGACGAAAACAC	191
PAIefs-79R	GCGCTACACTTACAACAATCC	
PAIefs-80F	AGAATTGATGATCGCTTGATCC	153
PAIefs-80R	CGAAGAAATTCCAGCAGGAAC	
PAIefs-81F	CCTGTTGTTTCTATGACGCC	200
PAIefs-81R	ACCATTCCCAAATTAACTCCAG	
PAIefs-82F	ACGATTTCGATGCTGCAAC	202
PAIefs-82R	TCATCTGTATCCCAGCCAAG	
PAIefs-83F	GGAGCTGATAATGCTTGTGC	202
PAIefs-83R	AAGAATTACCTGCTGCCAAC	
PAIefs-92F	GCTTTAGTTTCACTACAATCCCC	194
PAIefs-92R	GCGTCTACCTGCTTCTTAATTAC	
PAIefs-93F	GGGAACATATGCCGGAATG	293
PAIefs-93R	CATGATGCACAATTAGCACTG	
PAIefs-94F	GGCGATGCTATTTCTCATGC	223
PAIefs-94R	CGGTTGAGCTATTTGCAACAC	
PAIefs-119F	TCATCTCCTTCTTTCCCTGC	193
PAIefs-119R	AACGACTGATTACGCTTGAC	
PAIefs-123F	CCACCGAAGTTGAGTGTATC	198
PAIefs-123R	TCGATCTTCTGCTTCTTGTTC	
PAIefs-126F	CCAAACACGACTTCTTGCTC	198
PAIefs-126R	CACGGATTCACTGCTAAAGG	
PAIefs-129F	AGCCAATGAAACGACAGAAG	214
PAIefs-129R	CGGCTCAATACCAGAATTCC	
PAIefs-128-129 F	ATCAGGTCAAACGGATCAAA	197
PAIefs-128-129 R	CTGACAGAATGGCTCGTCTT	
PAIefs-125-F	CTAGGAATACAGCGACCAATAC	597
PAIefs-125-R	CCGACTTTGCTATCGGAAC	
PAIefs-126-F	TTTTGTCCAAGTGACATCGG	628
PAIefs-126-R	TTCAGAGATGGAAACAAGAGTG	
PAIefs-128F	AGGGACAGGGTTTTCGATTT	477
PAIefs-128R	ATTTGGTTCGCCTTCCTCTT	
oxiRed-F	AACTGGTTTGAACCGTGAGG	385
oxiRed-R	TTGCATCAATGGTGACCAAG	
